# Characterization and Antimicrobial Studies of Iturin-Like and Bogorol-Like Lipopeptides From *Brevibacillus* spp. Strains GI9 and SKDU10

**DOI:** 10.3389/fmicb.2021.729026

**Published:** 2021-10-29

**Authors:** Shelley Sardul Singh, Deepika Sharma, Piyush Baindara, Stanzin Choksket, Harshvardhan, Santi M. Mandal, Vishakha Grover, Suresh Korpole

**Affiliations:** ^1^CSIR-Institute of Microbial Technology, Chandigarh, India; ^2^Indian Institute of Technology, Kharagpur, India; ^3^Dr. Harvansh Singh Judge Institute of Dental Sciences and Hospital, Panjab University, Chandigarh, India

**Keywords:** *Brevibacillus*, iturin, bogorol, ILL, BLL, lipopeptide

## Abstract

Accession numbers for whole-genome sequence of *Brevibacillus* sp. strain GI9 and SKDU10 are CAGD01000001 to CAGD01000061 and LSSO00000000, respectively. Members of the genus *Brevibacillus* have been demonstrated to produce a variety of bioactive compounds including polyketides, lipopeptides and bacteriocins. Lipopeptides are non-ribosomally synthesized surface-active compounds with antimicrobial, antitumor, and immune-stimulatory activities. They usually exhibit strong antifungal and antibacterial activities and are considered as promising compounds in controlling fungal diseases. In this study, we have characterized two lipopeptides from *Brevibacillus* sp. strains GI9 and SKDU10. The corresponding lipopeptides were purified by reverse-phase high-performance liquid chromatography. Mass analysis and characterization by MALDI-TOF-MS (Matrix-assisted laser desorption ionization time-of-flight mass spectrometry) analysis revealed production of an iturin-like lipopeptide by strain GI9 and bogorol-like lipopeptide by strain SKDU10. Both lipopeptides exhibited broad spectrum antibacterial activity and inhibited the growth of various fungi. They showed minimum inhibitory concentration (MIC) values between 90 and 300 μg/ml against indicator strains of bacteria and drug-resistant *Candida* indicator strains. The lipopeptides did not show phytotoxic effect in seed germination experiments but caused hemolysis. Further, both lipopeptides inhibited the growth of fungi on fruits and vegetables in *in vitro* experiments, thereby exhibited potential use in biotechnological industry as effective biocontrol agents.

## Introduction

The ecological balance of soil microbes has been disturbed due to the extensive use of chemicals (fertilizers or pesticides) in order to increase crop productivity, control plant diseases, and increase global food security (Bargaz et al., 2018; Singh et al., 2019a,b). Alteration in ecological balance has led to the development of resistant strains of pathogens, causing severe plant diseases and increased health hazards to humans (Gill and Garg, 2014; Velásquez et al., 2018). To control the use of commercially available chemical fungicides including dicarboximides, carbamates, benzimidazoles, and triazoles, biocontrol agents need to be developed (Coates and Hu, 2007). Currently, antimicrobial peptides (AMPs) including lipopeptides and glycopeptides have been widely studied as potential antibacterial and antifungal agents (Lei et al., 2019). Lipopeptides, such as surfactins, iturins, and fengycins, are well studied for their potent antagonistic activities against various phytopathogens. Therefore, lipopeptides are considered as potential alternatives to the growing problem of resistance to the conventional chemicals, fungal infections, and life-threatening diseases (Meena and Kanwar, 2015; Chen and Lu, 2020).

Members of genus *Brevibacillus* are widely spread in nature and have been reported as rich source of many antimicrobial peptides (Singh et al., 2015; Baindara et al., 2016; Yang and Yousef, 2018). They synthesize AMPs through ribosomal as well as non-ribosomal pathways. Ribosomally produced peptides from *Brevibacillus* include laterosporulins, Bac-GM100, etc. (Singh et al., 2012; Yang and Yousef, 2018). Non-ribosomal pathway yields lipopeptides and glycopeptides, which may be further categorized based on the presence of lipid chain and cyclization. Lipopeptides are widely produced by members of various genera such as *Bacillus*, *Brevibacillus, Paenibacillus, Pseudomonas*, and *Streptomyces* (Grady et al., 2016; Manhas and Kaur, 2016). They are structurally highly diverse and have the ability to decrease the surface as well as interfacial tension (Hu et al., 2019). The number of amino acids varies from 7 to 25 between various groups of biosurfactant lipopeptides. Each group of biosurfactants exhibits a peptide backbone, which is essentially attached with a fatty acid chain that varies from 13 to 17 carbon atoms. The amino acid composition of peptides display variation in the chemical structure and as a result, they exhibit differences in toxicity to non-target organisms and resistance in adverse conditions (Raaijmakers et al., 2010). These properties qualify them as suitable green and eco-friendly biocides in comparison to their synthetic counterparts for managing pathogens in the agriculture and food industry (Nitschke and Costa, 2007; Shekhar et al., 2015; Malviya et al., 2020).

The biosurfactant lipopeptides also include therapeutically known compounds like gramicidin, gramicidin S, tyrocidine, brevibacillin brevistin, tostadin, and spergualin that were shown to act on the cell membrane. Apart from antimicrobial activity, these lipopeptides were also known for antitumor and antiviral activities (Peypoux et al., 1999). However, intrinsic toxicity and low stability of lipopeptides are major hurdles for their utilization as novel therapeutics (Raaijmakers et al., 2010). Consequently, such peptides can be used for agro-industrial purposes as there is an urgent need to develop new biocides that can help in controlling plant diseases and to reduce the microbial infection in harvested crops. To this effect, AMPs produced by members of the genus *Brevibacillus* were used as potential food preservatives and feed additives (Yang and Yousef, 2018). Thus, in this study, we have characterized two antimicrobial lipopeptides produced by *Brevibacillus* sp. strains GI9 and SKDU10.

## Materials and Methods

### Bacterial Strains and Media

*Brevibacillus* sp. strains GI-9 and SKDU10 were known to produce class IId bacteriocins identified as laterosporulins a (Singh et al., 2012; Baindara et al., 2016). Both strains were grown on nutrient agar (NA, Himedia, India). All test strains used in this study were obtained from Microbial Type Culture Collection and Gene bank (MTCC), Chandigarh, India. Clinical *Candida* strains were obtained from the National Culture Collection of Pathogenic Fungi (NCCPF), PGIMER, Chandigarh, India, and all strains were grown on recommended media.

### Production and Purification of Antimicrobial Compounds

Laterosporulin producing *Brevibacillus* strains GI9 and SKDU10 were cultivated in nutrient broth (NB, Himedia) for lipopeptide production. Briefly, 2% (v/v) of the overnight grown culture of GI9 and SKDU10 was inoculated to 1 L of NB medium and incubated at 30°C on a rotary shaker (innova44, New Brunswick Scientific). After 48 h incubation, the broth was centrifuged at 8000 rpm for 20 min to produce cell-free broth. Antimicrobial compounds were extracted using the solvent extraction technique. Equal volumes of ethyl acetate and cell-free broth were mixed, and subsequently, the solvent phase was recovered using a separating funnel. Ethyl acetate was evaporated using a rotary evaporator (BUCHI Rotavapor R-200) and the residues were redissolved in methanol. Methanol was again removed and extracts dissolved in water were tested against *Staphylococcus aureus* MTCC 1430 and *Candida albicans* MTCC 183 to screen antimicrobial activity. For purification, solvent extracts were subjected to reverse-phase high performance liquid chromatography (HPLC) (1260 Infinity, Agilent Technologies, United States) with a semi-preparative C18 column (Pursuit 10C18 250 × 21.2 mm) using water and acetonitrile as solvents containing 0.12 and 0.1% trifluoroacetic acid (TFA), respectively. Acetonitrile gradient was run through the column for a duration of 55 min program as follows: 0–60% for 0–45 min, 60–80% for 45–50 min, and 80–100% for 50–55 min. The elution from the column was monitored at 220 nm and all the peaks were collected. Acetonitrile and water mixture was removed using a speed vacuum (Eppendorf, United States). Residues were dissolved in water and screened for antimicrobial activity. Peaks with antimicrobial activity were pooled after multiple HPLC runs and subjected to freeze drying. Lyophilized powder was stored until further use before dissolving in water.

### Identification of Active Compounds by Matrix-Assisted Laser Desorption Ionization

Purified active compounds were used to determine mass by Matrix-assisted laser desorption ionization (MALDI). For this, the lyophilized compound was re-suspended in water and mixed with matrix alpha-cyano-4-hydroxycinnamic acid (CHCA, 10 mg/ml) in 1:1 ratio. The mixture solution (2.0 μl) was spotted onto the MALDI 100 well stainless steel sample plate and allowed to air dry. Spectra were recorded in positive ion linear mode using 337 nm N_2_ laser operated in accelerating voltage 20 kV (Applied Biosystem, United States). Reproducibility of the spectrum was checked several times from separately spotted samples. MS/MS sequencing of the peptide was carried out following Hakomori’s methylation procedure (Hakomori, 1964) after cleavage of lactone ring with 10% NaOH in CH_3_OH at room temperature for 16 h. The spectra were recorded in the post-source decay (PSD) ion mode as an average of 100 laser shots with a grid voltage of 75%. The reflector voltage was reduced in 25% steps and guide wire was reduced 0.02–0.01% with an extraction delay time of 100 ns.

### Gas Chromatography-Mass Spectrometry for Fatty Acid Analysis

Acid hydrolysis of purified lipopeptides was performed with 5 mg of each peptide that was added with 0.5 ml of 6 M HCl and incubated at 90°C for 18 h in sealed tubes. The fatty acids were extracted with ether and esterified with methanol in a solution containing 0.95 ml and 0.05 ml of 98% H_2_SO_4_ by incubating at 65°C for 6 h. Fatty acid methyl esters were obtained after extraction with n-hexane and analyzed by gas chromatography-mass spectrometry (GC-MS) using Clarus 500 GC (PerkinElmer, United States). Helium was used as carrier gas at a flow rate of 1.0 ml/min. The column temperature was maintained at 120°C for 3 min and then gradually increased (8°C/min) to 260°C.

### Analysis of Biosynthetic Gene Clusters Encoding Secondary Metabolites

The genomic analysis for the detection of the biosynthetic gene cluster (BGC) is analyzed using the antiSMASH database for identification of secondary metabolite or lipopeptide biosynthesis clusters (Medema et al., 2011; Blin et al., 2019). The genome sequence of both *Brevibacillus* spp. strains GI9 (CAGD01000001 to CAGD01000061) and SKDU10 (LSSO00000000) were uploaded at antiSMASH^1^ for an automated prediction with a specific focus on NRPS and PKS gene clusters.

### Determination of Antimicrobial Activity and Minimum Inhibitory Concentration Values

The antimicrobial activity of HPLC purified compounds was determined against various test strains including *S. aureus* MTCC 1430, *Vibrio cholera* MTCC 3904, *Bacillus subtilis* MTCC 121, *Listeria monocytogenes* MTCC 839, *C. albicans* MTCC 183, *C. albicans* MTCC 1637, *C. albicans* MTCC 227, *C. tropicalis* MTCC 184, *C. glabrata* MTCC 3019, and *C. inconspicua* MTCC 1074. Antimicrobial activity was also tested against clinical *Candida* strains and phytopathogenic fungi *viz*. *Fusarium moniliforme* MTCC 158, *Alternaria brassicicola* MTCC 2102 and *Colletotrichum acutatum* MTCC 1037. Bacterial and *Candida* test strains were grown in NB and yeast malt broth (YMB), respectively. Fungal test strains were grown in potato dextrose broth (PDB). Minimum inhibitory concentrations (MICs) of purified compounds were evaluated against test organisms by using a microtiter plate dilution assay. Mid-log-phase test strains (5 × 10^5^ CFU/ml) were incubated in a 96-well plate with different concentrations of the lipopeptides for 24–48 h at 37°C (final volume of 200 μl). OD was measured at 600 nm after 24 and 48 h for bacteria and yeast, respectively, using an ELISA microplate reader (Thermo Scientific, United States). MIC against phytopathogenic fungi was determined using the 96-well microtiter plate method (Sharma et al., 2020). The lowest concentration that did not show growth was considered as MIC.

### Thin Layer Chromatography Bioautography Assay

Overlay thin-layer chromatography (TLC)-bioautography assay was used to determine the antimicrobial activity of the purified antimicrobial compounds (Wedge and Nagle, 2000). For bioautography, the antimicrobial compounds (10 μg/ml) were loaded on TLC silica gel plates in duplicates and were developed in one dimension using a solvent system containing chloroform: methane: water (65:12:4 v/v). TLC plate was cut into two vertical parts after running. One part of the TLC was used for direct detection of antimicrobial activity by overlaying it on NA containing *S. aureus* MTCC 1430 (about 10^6^ cells ml^–1^). After 1 h diffusion at 4°C, the TLC silica plate was removed and the NA plate was incubated for 24 h at 37°C. Other part of the TLC was sprayed with phosphomolybdic acid as a staining reagent for lipids.

### Collapse Drop Assay and Determination of Critical Micelles Concentration

Collapse Drop Assay (CDA) was performed as mentioned earlier (Sharma et al., 2020). To a well of 96 well microtiter plate, 2 μl mineral oil was added and equilibrated at 37°C for 24 h. Purified lipopeptide (5 μl of 10 μg/ml stock) was applied to the top of oil droplet and its shape was observed after 1 min with a magnifying glass for collapse and spread due to reduction in surface tension. Water and triton X-100 served as controls (Youssef et al., 2004). To determine critical micelle concentration (CMC), different concentrations of purified lipopeptides were prepared and surface tension was measured on a tensiometer (KRUSS K10T, Germany) at room temperature. The values for CMC were estimated by plotting surface tension as a function of concentration of lipopeptides. A point of intersection was located between the pre and post CMC data plot. The mean value of triplicate experiments was used to increase the accuracy. Concentrations ranging from 0.1 to 1000 μg/ml were used to determine surface tension on a tensiometer.

### Fourier Transform Infrared Spectroscopy

Fourier Transform Infrared Spectroscopy (FTIR) was performed using Perkin Elmer Spectrum RX-IFTIR (United States) spectrophotometer. Pure lyophilized lipopeptides (5 mg) were mixed with 100 mg Potassium bromide to obtain a pellet. Spectra were collected over the range of 4000 to 650 cm^–1^. Average of 50 scans over the entire range were taken and data analyzed.

### Time Kill Assay

Killing kinetics of purified compounds was performed using cells of *S. aureus* MTCC 1430, *V. cholerae* MTCC 3904, and *C. albicans* MTCC 183. Test strains were grown to obtain OD between 0.2 and 0.3 (OD 600 nm), centrifuged, and washed thrice with sterile PBS. Pellets were suspended in PBS and treated with different concentrations of active compounds. Cells after treatment were washed, resuspended, and serially diluted cell suspensions were plated on NA plates. After overnight incubation at 37°C, CFU were recorded (Singh et al., 2021).

### Effect of pH, Temperature and Hydrolytic Enzymes on Peptides Activity

Sensitivities of purified antimicrobial compounds to temperature, pH, and hydrolytic enzymes were confirmed by performing well-diffusion assay. pH stability was determined by adjusting the pH of aliquots of purified antimicrobial compounds from 2.0 to 12.0 (using 10 mM HCl or NaOH) and incubating at room temperature for 4 h. The residual activity was measured after neutralizing the sample to pH 7.0. To determine temperature susceptibility, antimicrobial compounds were incubated at different temperatures ranging from 37 to 100°C for 1 h and 121°C for 15 min, and then residual antimicrobial activity was determined after cooling the samples to room temperature. Similarly, proteolytic enzymes such as trypsin and proteinase K were used at three different concentrations (0.1, 1.0, and 5.0 mg/ml) to ensure their effect on the activity of antimicrobial compounds. The enzyme solutions were prepared in 50 mM phosphate buffer (pH 7.0). All reactions were performed at 37°C for 6 h followed by deactivation of the enzyme by heating the solution in boiling water for 5 min before performing the activity assay.

### Toxicity Testing

For hemolysis assay rabbit erythrocytes were treated with different concentrations of purified active antimicrobial compounds ranging from 250 to 750 μg/ml and incubated at 37°C for 24 h in a 5% CO_2_ incubator. The release of hemoglobin was monitored by measuring the absorbance at 540 nm in a microplate reader. PBS and 0.1% Triton X-100 were used as controls for 0 and 100% hemolysis, respectively (Singh et al., 2021). For phytotoxicity assay, sterilized *Vigna radiata* (Moong) seeds were treated with different concentrations of purified antimicrobial compounds (250–750 μg/ml). Seeds soaked in water and 0.1% mercuric chloride were used as negative and positive control, respectively. Seeds germination was observed up to 3 days of incubation.

### Effect of Antimicrobial Compounds on Cell Membrane of Pathogens

Effect of antimicrobial compounds on cell membrane of test organisms was determined by transmission electron microscopy (TEM). Test strains, *S. aureus* MTCC 1430, *V. cholerae* MTCC 3904, and *C. albicans* MTCC 183 were cultured to mid-log phase, washed, and re-suspended in 1X PBS (OD 600 nm 0.2). The cell suspensions were incubated (37°C) with 1X MIC of purified antimicrobial compounds for 30 min. Cells after treatment were centrifuged, washed twice with PBS. For TEM, cells of *S. aureus* MTCC 1430 and *V. cholerae* MTCC 3904 were subjected to negative stain with 0.1% (w/v) sodium phosphotungstate (PTA, Sigma, United States) in water for 1 min, on a carbon-coated copper grid with 300 mesh (Polyscience, United States) and observed under transmission electron microscope (JEOL JEM 2100, United States) (Raje et al., 2006). For SEM, *C. albicans* MTCC 183 cells were immobilized on poly L-lysine glazed glass slides and incubated at 4°C for 2 h. Cells were fixed using Karnovsky’s fixative and serially dehydrated with gradient of ethanol, freeze dried, and platinum coated. ZEISS EV040 (United States) scanning electron microscope was employed to photograph the cells (Raje et al., 2006). Spores of *A. brassicicola* MTCC 2102 were treated with 2X MIC of lipopeptides for 2 h and observed under phase-contrast microscope (Nickon H600L, Japan). Untreated bacterial cells and fungal spores were used as controls.

### Protection Ability of Lipopeptides Against Spores of *Alternaria brassicicola* Microbial Type Culture Collection 2102

The surface of the tomatoes and grapes was sterilized with 5% NaOCl for 5 min and rinsed three times with plenty of sterile water. After sterilization, tomatoes were cut into slices and wounds of 3 mm were inflicted with a sterile scalpel on the surface of the grapes. Tomato slices and grapes were divided into four groups. Contents of group 1 were treated with water only (negative control). Contents of group 2 were treated with 50 μl of a spore suspension (spores 10^7^ conidia per ml) of *A. brassicicola* MTCC 2102 (positive control without treatment). Contents of group 3 were first treated with 500 μl of 2 mg/ml solution of purified lipopeptides separately and after adsorption of lipopeptides, fungal spores were added. Contents of the fourth group were treated with purified lipopeptides only without addition of fungal spores. Tomato slices and grapes from all four groups were incubated at 25°C under controlled humidity conditions for 4 days and observed. Each treatment was performed with three biological and technical replicates.

### Statistical Analysis

All experiments were performed in triplicates and the data presented as mean ± standard deviation (SD). Student’s *T*-test was used to calculate statistical significance (*p*-value < 0.05 considered significant; ^∗^*p* < 0.05, ^∗∗^*p* < 0.005, and ^∗∗∗^*p* < 0.0005).

## Results

### Characterization of Antimicrobial Compounds

**FIGURE 1:**
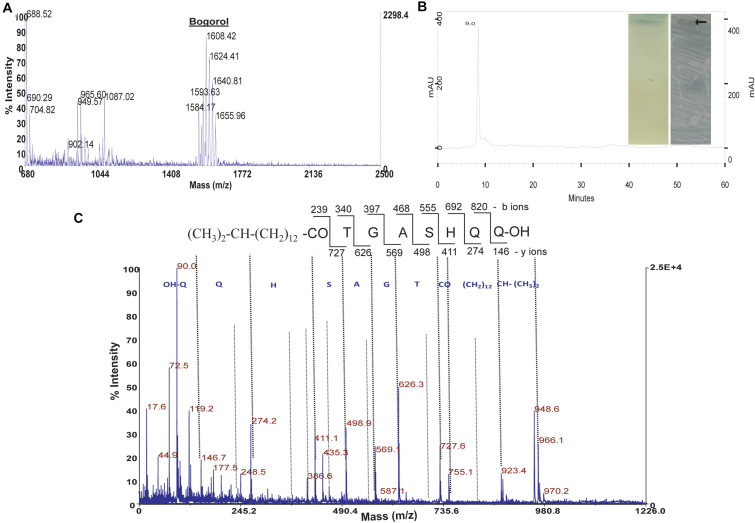
Purification and characterization of antimicrobial compound from strain GI9. **(A)** MALDI of crude extract from strain GI9 showing secretion of different compounds. **(B)** HPLC chromatogram of purified antimicrobial peptide at retention time 9 min. Inset shows phosphomolybdic acid stained TLC of pure compound and bioautogram with inhibition zone against *S. aureus* MTCC 1430. **(C)** MS/MS fragmentation pattern of iturin-like lipopeptide (ILL) at *m/z* 966.1 Da.

The antimicrobial substances from strains GI9 and SKDU10 were produced in large quantity by growing them in NB medium under optimal conditions. The antimicrobial substances were extracted using ethyl acetate. Solvent extracts of both strains GI9 and SKDU10 displayed antimicrobial activities against various indicator strains. The crude extract obtained by strain GI9 showed multiple major peaks when subjected to MALDI (Figure 1A). A cluster of peaks ranging from *m/z* 1584 to 1640 corresponded to different analogs of bogorol family (bogorol A–E). Another major peak was observed at *m/z* 1087 corresponding to a member of surfactin family. A peak at *m/z* 965.6 was found, however, this molecular weight did not match with other lipopeptides classified under iturins or surfactins. We hypothesized that this peak corresponded to a novel compound and isolated this compound from crude extract. The compound was purified using reverse-phase HPLC that showed a peak at a retention time of 9 min at 220 nm (Figure 1B) suggesting the presence of peptide bonds. The collected peak possessed broad spectrum activity against bacteria and *Candida*. The compound produced single band on TLC that stained bluish black with phosphomolybdic acid indicating its lipid nature. When subjected to TLC-based bioautography, the band corresponding to pure lipopeptide inhibited *S. aureus* MTCC 1430 (inset of Figure 1B). HPLC purified lipopeptide was investigated for *de novo* sequence through MS/MS analysis, which yielded mass peaks at *m/z* 119, 274, 411, 498, 569, 626, and 727 that represented loss of amino acid residues (Figure 1C) and unveiled the primary structure as Asp-Asp-His-Ser-Ala, Gly-Thr, and a fatty acid as shown in the inset of Figure 1C. The Mass accuracy was calculated as (measured-theoretical)/theoretical mass × 10^6^ ppm and mass error values are given in Supplementary Table 1. The fatty acid moiety had a β-hydroxy fatty acid containing 14 Carbon molecules. Iturin biosurfactants usually contain seven amino acid residues along with a fatty acid chain and accordingly we hypothesize that the antimicrobial lipopeptide in this study represents a novel iturin like lipopeptide (ILL).

**FIGURE 2:**
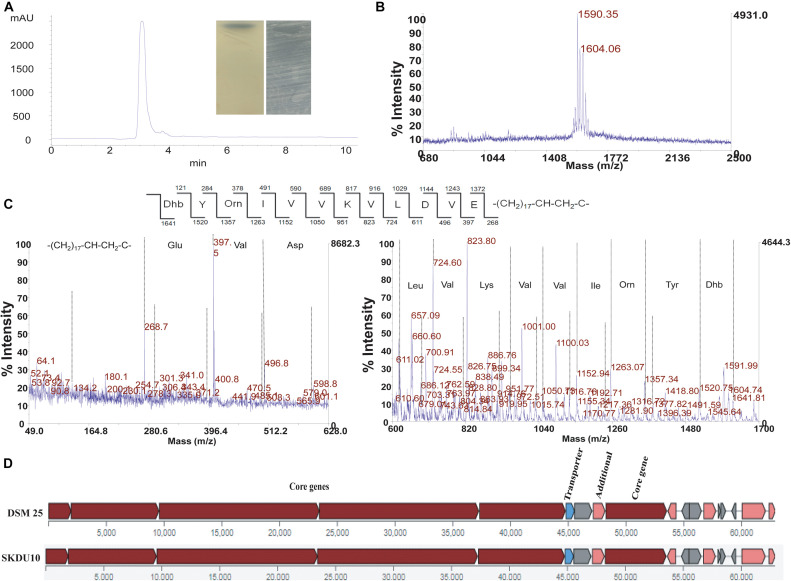
Purification and characterization of antimicrobial compound from strain SKDU10. **(A)** HPLC chromatogram purified peptide at retention time 3 min. Inset shows phosphomolybdic acid-stained TLC of pure compound and bioautogram with inhibition zone against *S. aureus* MTCC 1430. **(B)** MALDI spectrum showing intact mass of peptide *m/z* 1604 Da. **(C)** MS/MS fragmentation pattern of bogorol like lipopeptide (BLL) at *m/z* 1604 Da. **(D)** Biosynthetic gene cluster from strain SKDU10 consisting of modules responsible for secretion of BLL in comparison to bogorol biosynthetic cluster of *B. laterosporus* strain DSM25.

Crude extract obtained by strain SKDU10 was purified by HPLC showed a single peak at 220 nm with retention time 3 min (Figure 2A). The collected peak showed a single band on TLC silica plate confirming the purity of the peptide. The band turned bluish black on phosphomolybdic acid staining and was found to be active against *S. aureus* MTCC 1430 in bioautography (inset of Figure 2A). Together these data suggested that the active antimicrobial compound is a lipopeptide. During MALDI analysis, the intact mass was found to be *m/z* 1604.06 (Figure 2B). Another mass peak with an interval of 14 Da observed in mass spectrum of lipopeptides indicates a different number of methyl groups (CH_2_) associated with the fatty acid chain. The lipopeptide was further subjected to *de novo* sequencing and MS/MS fragmentation pattern for parent peak at *m/z* 1604, which revealed the primary structure of the peptide sequence as Dhb-Tyr-Orn-Ile-Val-Val-Lys-Val-Leu-Asp-Val-Glu (Figure 2C). The mass error values are given in Supplementary Table 2. The peak obtained at *m/z* 268.7 can be attributed to C17 hydroxy fatty acid side chain. Amino acid composition of lipopeptide matched with bogorol analogs with some variations and thus mining of the genome sequences was performed using antiSMASH database. Bogorol encoding NRPS clusters were observed in genomes of strains GI9 and SKDU10 with 100% and 72% identity, respectively (Table 1). Identification of novel BGCs or homology search performed using profile hidden Markov model (pHMM) revealed an E-value ranging between 1.6e-79 and 9.9e-103 for strain GI9 and it was 2.3e-105 to 9.4e-106 for strain SKDU10. Bogorol producing strain *B. laterosporus* DSM 25 was used as reference genome (E-value range 1e-111 to 8.3e-79) with pHMM parameters aiming at PP-binding or NAD_binding, AMP-binding domains, condensation domain, and AMP-dependent synthatase and ligase. The cluster essentially contained biosynthetic and associated genes encoding adenylation(A), thiozole formation (T) condensation (C), epimerization (E), and termination (TE) modules (Figure 2D) with high identity. The purified lipopeptide was named bogorol like lipopeptide (BLL). In addition, positive reaction in CDA for HPLC purified ILL and BLL from strains GI9 and SKDU10, respectively, also confirmed their lipopeptide nature. The CMC for ILL and BLL was found to be 90 and 110 μg/ml, respectively.

**TABLE 1:** Genome search results showing putative biosynthetic clusters encoding known antimicrobial substances analyzed by antiSMASH bioinformatics tool.

Compound	Mol. wt.	Gene composition	Strain GI9			Strain SKUDU10			References
			
			Nucleotide sequence identity (%)	Region	Nucleotide seq (From to)	Nucleotide sequence identity (%)	Region	Nucleotide seq (From to)	
Tyrocidine	1270.5	NRPS	12	NZ_CAGD010000017.2	52,248–99,261	12	NZ_LSSO0100041.1	1–59,439	Lipmann et al., 1941
Bogorol A	1584	NRPS	100	NZ_CAGD01000001.1	263,605–358,538	72	NZ_LSSO0100074.1	1–59,851	Dejong et al., 2016
Obafluorin	358.3.	NRPS	14	NZ_CAGD01000004.1	1–96,545	–			Scott et al., 2017
Tauramamide	864	NRPS	36	NZ_CAGD01000002.1	45,343–174,440	9	NZ_LSSO0100061.1	1–21,011	Dejong et al., 2016
Bacillibactin	882.789	NRPS	15	NZ_CAGD01000003.1	103,400–199,283	–			Kunst et al., 1997
Brevicidine	507	NRPS	100	NZ_CAGD01000002.4	348,872–438,222	63	NZ_LSSO0100085.1	1–56,502	Li et al., 2018
Petrobactin	718.8	Siderophore	100	NZ_CAGD01000001.2	421,376–435,075	100	NZ_LSSO0100071.1	7,913–21,612	Cendrowski et al., 2004
Basiliskamide A/B	385.5	Polyketide	90	NZ_CAGD010000027.1	1–60,445	9	NZ_LSSO01000152.1	1–6,065	Theodore et al., 2014
Chejuenolide	388.5	NRPS and polyketide	7	NZ_CAGD01000002.2	177,888–247,190	7	NZ_LSSO0100064.1	1–22,844	Ng et al., 2018
Zwittermicin	396.4	NRPS and plyketide	11	NZ_CAGD010000017.1	184–49,900	44	NZ_LSSO01000100.1	1–65,093	Milner et al., 1996
Merosterol	583.497	Sterol/terpene	–			10	NZ_LSSO01000158.1	1–36,882	Moosmann et al., 2017

### Fourier Transform Infrared Spectroscopy Analysis

Fourier Transform Infrared Spectroscopy (FTIR) spectra of purified lipopeptides exhibited absorbance peak at wavenumbers 3400 cm^–1^ to 3200 cm^–1^ as a result of C-H, N-H stretching vibrations and intramolecular hydrogen bonding attributed to carbon-containing compounds with amino groups (Supplementary Figure 1). Absorbance at 3000 cm^–1^ to 2800 cm^–1^ indicated the presence of aliphatic chain and C-CH_3_ bonding. The peak with maximum absorbance was observed at 1700 cm^–1^ to 1600 cm^–1^ due to stretching of CO-N bond of peptide molecule. Absorbance at 1550 cm^–1^ to 1520 cm^–1^ is due to the deformation of N-H bond coupled with C-N stretching. Peaks in this region also signify the presence of C = O bonds.

### Minimum Inhibitory Concentration and Killing Kinetics

**FIGURE 3:**
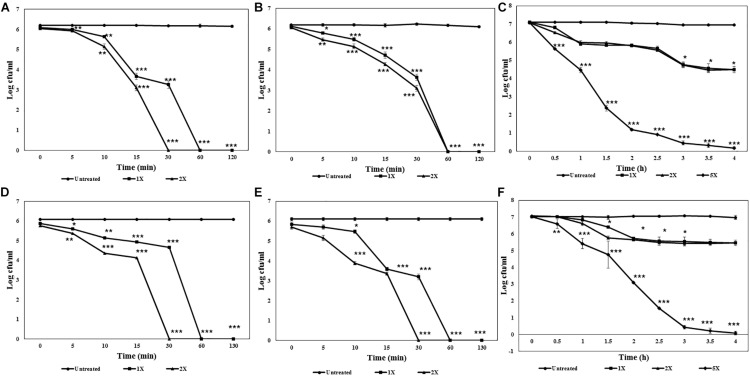
Killing kinetics of antimicrobial lipopeptides. **(A)** ILL against *S. aureus* MTCC 1430. **(B)** ILL against *V. cholerae* MTCC 3904. **(C)** ILL against *C. albicans* MTCC 1430. **(D)** BLL against *S. aureus* MTCC 1430. **(E)** BLL against *V. cholerae* MTCC 3904. **(F)** BLL against *C. albicans* MTCC 1430.

HPLC purified lipopeptides from strains GI9 and SKDU10 had broad spectrum antimicrobial activity. ILL and BLL exhibited MIC values ranging from 90 to 500 μg/ml against various indicator strains (Table 2). Both lipopeptides inhibited all test strains pertaining to bacteria, filamentous fungi, and *Candida* including clinical isolates. Additionally, both lipopeptides were active against fluconazole-resistant strains of *Candida* and filamentous fungi with MIC between 150 and 300 μg/ml (Table 2). Kinetic studies of ILL showed complete reduction in population of *S. aureus* MTCC 1430 within 30 min using 2X MIC treatment and 60 min with 1X MIC treatment (Figure 3A). There was a complete reduction in the load of *V. cholerae* MTCC 3904 within 60 min upon treatment with 1X and 2X MIC of ILL (Figure 3B). Complete killing of *C. albicans* MTCC 183 was achieved after 4 h incubation with 5X MIC of ILL and 2 log reduction in growth was observed with 1X and 2X MIC (Figure 3C). Similarly, BLL caused a complete reduction of bacterial cells of *S. aureus* MTCC 1430 and *V. cholerae* MTCC 3904 within 30 min upon 2X MIC treatment and 30 min upon 1X MIC treatment (Figures 3D,E). Complete killing of cells of *C. albicans* MTCC 183 was not observed with 1X and 2X MIC within 4 h, however, 5X MIC of BLL killed all population within 4 h (Figure 3F). No reduction in CFU/ml count was observed for the untreated cells.

**TABLE 2:** Determination of minimum inhibitory concentrations (MIC) against various indicator and clinical strains by ILL and BLL (concentration range of lipopeptides tested was 10–550 μg/ml with 5 μg/ml increments).

Test organism	Reference number	MIC value (μg/ml)			
		Fluconazole*	Amphotericin B*	BLL	ILL
**Bacteria**					

*S. aureus*	MTCC 1430	–	–	140–150	150–170
*L. monocytogenes*	MTCC 839	–	–	230–240	200–220
*B. subtilis*	MTCC 121	–	–	225–235	260–270
*V. cholerae*	MTCC 3904	–	–	140–150	105–115

**Yeast**					

*C. tropicalis*	MTCC 184	R	35	290–310	150–160
*C. glabrata*	MTCC 3019	R	25	290–300	190–210
*C. haemulonii*	MTCC 2766	R	100	340–360	300–320
*C. inconspicua*	MTCC 1074	50	10	100–110	140–160
*C. albicans*	MTCC 183	20	5	150–170	90–100
*C. albicans*	MTCC 1637	R	5	250–260	140–160
*C. albicans*	MTCC 227	R	5	300–310	140–150

**Clinical strains**					

*C. albicans*	400054	R	5	240–260	90–110
*C. tropicalis*	420024	R	50	300–320	190–210
*C. parapsilosis*	450030	10	50	290–310	150–160
*C. haemulonii*	470125	R	50	245–255	140–160
*C. auris*	470126	R	5	140–160	90–100
*C. rugosa*	470141	5	5	240–250	40–60
*C. kefyr*	410004	R	50	190–210	150–160
*C. krusei*	440009	1	5	240–250	100–120

**Fungi**					

*C. acutatum*	MTCC1037	R	R	430–450	390–410
*F. moniliforme*	MTCC 158	10	50	490–510	490–520
*A. brassicicola*	MTCC 2102	R	50	500–520	430–480

*Antifungal compounds with no activity against bacteria; R = resistant.

### Microscopic Examination of Indicator Strains After Treatment With Lipopeptides

**FIGURE 4:**
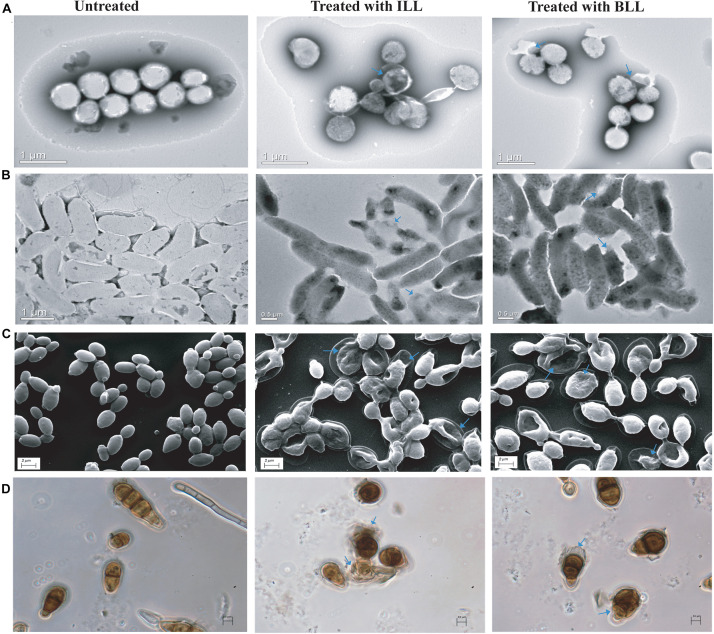
Microscopic examination of indicator strains after treatment with lipopeptides**. (A)** TEM micrographs of untreated and treated cells of *S. aureus* MTCC 1430. **(B)** TEM micrographs of untreated and treated cells of *V. cholerae* MTCC 3904. **(C)** SEM micrographs of untreated and treated cells of *C. albicans* MTCC 183. **(D)** Phase-contrast images of untreated and treated spores of *A. brassicicola* MTCC 2102. (Cell damage is marked by arrows).

To understand the killing ability of lipopeptides, treated indicator strains were observed under both electron and phase-contrast microscopy. The untreated *S. aureus* MTCC 1430, *V. cholerae* MTCC 3904, and *C. albicans* MTCC 183 cells showed smooth cell surface and appeared to be healthy and intact (Figures 4A–C). In contrast, ILL-treated cells showed lysis with accumulation of debris on cell surface, altered morphology, and shrinkage. Similarly, BLL treated cells also showed irregularities in structure with rupturing of cell wall and lysis (Figures 4A–C). Untreated spores of *A. brassicicola* MTCC 2102 showed complete cell membrane and homogeneous cytoplasm. Spores treated with ILL and BLL displayed structural irregularity in comparison to untreated cells. Treatment with lipopeptides showed disintegrated cells resulting in dispersion of the intracellular contents (Figure 4D).

### Temperature and Enzyme Sensitivity

Lipopeptides were found to be highly thermostable as both retained activity even after exposing to 121°C for 15 min (autoclaving). They were also stable between pH 4.0 and 10.0 and displayed antimicrobial activity. Studies on tolerance of the lipopeptides to proteolytic enzymes revealed both ILL and BLL were resistant to trypsin and proteinase K treatment. Additionally, lipopeptides were found to be completely stable to lipase treatment (Supplementary Table 3).

### Phytotoxicity and Hemolysis

**FIGURE 5:**
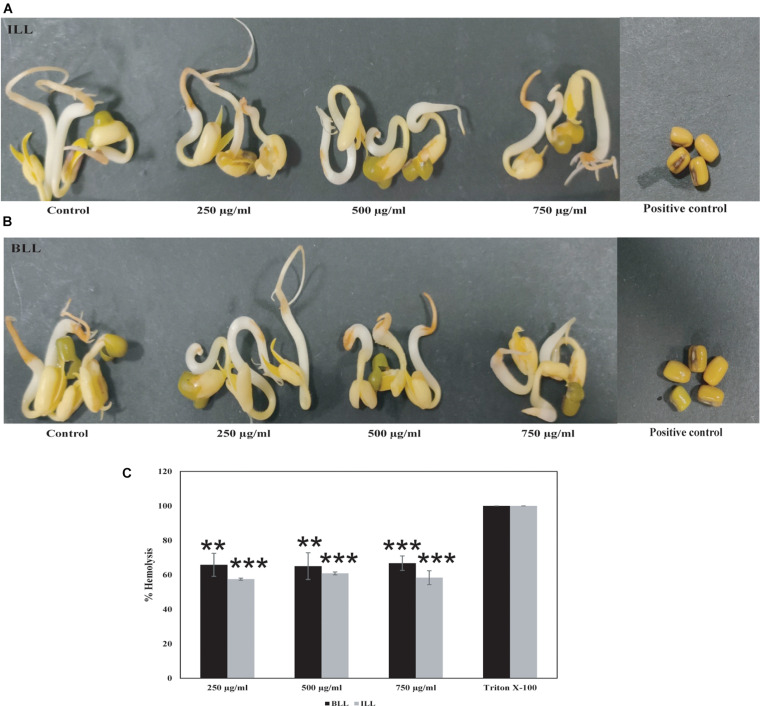
Toxicity testing of antimicrobial lipopeptides. Seed germination-based phytotoxicity of **(A)** ILL **(B)** BLL using *Vigna radiata*. Water and 0.1% mercuric chloride were used as negative and positive control, respectively. **(C)** Hemolysis assay showing lysis of erythrocytes by both lipopeptides.

ILL and BLL did not exhibit seed germination-based phytotoxicity against *Vigna radiata* (Moong) seeds at 750 μg/ml concentration (Figures 5A,B). The germination and seedling formation in test seeds and seeds soaked in water were similar and there was no variation in morphology or growth pattern. No germination was observed in seeds soaked in 0.1% mercuric chloride (Figures 5A,B). They showed hemolytic activity at 250 μg/ml. BLL exhibited more than 60% hemolysis as compared with triton X-100. About 55% hemolysis was also observed in ILL (Figure 5C).

### Protection of Tomato and Grape Slices From Fungi

**FIGURE 6:**
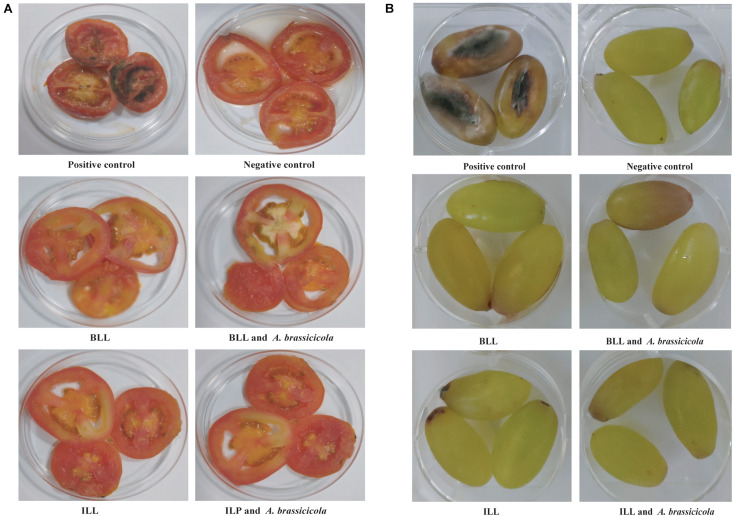
Protective ability of lipopeptides against infection of *A. brassicicola* MTCC 2102 in *in vitro* assay. **(A)** Inhibition of *A. brassicicola* MTCC 2102 on sliced tomato. **(B)** Inhibition of *A. brassicicola* MTCC 2102 on grapes.

Protective ability of lipopeptides to increase the shelf-life of tomato and grape was also checked. Slices were inoculated with spores of *A. brassicicola* MTCC 2102. Fungal growth was observed on slices in positive control group after 2 days of incubation and increased thereafter. In contrast, no fungal growth was observed on the surface of slices treated with ILL and BLL sprayed with spores of *A. brassicicola* MTCC 2101 (Figures 6A,B). Furthermore, slices treated with only lipopeptides and only water (negative control) did not show any adverse effects on morphology and texture.

## Discussion

Members of the genus *Brevibacillus* are known to produce bioactive molecules with antimicrobial, insecticide, pesticide, and nematicide activities. The antimicrobials from members of the genus *Brevibacillus* include antibiotics, antimicrobial peptides, and lipopeptides (Desjardine et al., 2007; Singh et al., 2012; Zhao and Kuipers, 2021). Lipopeptides are typically composed of lipid chains attached to a peptide and display strong antifungal activity (Makovitzki et al., 2006). Strains of *Brevibacillus* are well known for antimicrobial peptides with activity against bacteria and plant pathogenic fungi (Orlova et al., 1998; Singh et al., 2015; Baindara et al., 2016; Yang and Yousef, 2018). The strong inhibition and selective spectrum exhibited by some of these antimicrobial substances have attracted the researchers to exploit them for applications like antibiotic, biocontrol agents and food additives or preservatives (Cleveland et al., 2001; Song et al., 2012, 2013). Therefore, in this study, we have identified two lipopeptides by mass spectrometry and investigated their antimicrobial activity against various bacteria, yeast, and filamentous fungi for their plausible applications.

Solvent extracts from both strains displayed promising antibacterial, anticandidal and antifungal activities. Further, MALDI analysis allowed molecular weight-based identity with different analogs of bogorol, a surfactin, and an unknown low molecular weight substance. This is in accordance with earlier observations on production of different bioactive lipopeptides by individual strains of *Bacillus* where co-production of surfactins, antibiotics, lipopeptides, and bacteriocin was reported (Tsuge et al., 1996; Roongsawang et al., 2002; Sharma et al., 2020). Moreover, *Bacillus* sp. were reported for simultaneous production of lipopeptides like iturin, surfactin, kurstakins, and fengycin families, which were identified and characterized by MALDI-MS analysis (Asaka and Shoda, 1996; Vater et al., 2002; Koumoutsi et al., 2004; Fanaei et al., 2021). In fact, lipopeptides including different analogs of bogorol were elucidated based on their structure determined by analyzing degradation products obtained in ESI-MS/MS (Barsby et al., 2006; Zhao and Kuipers, 2021). Initially, the detailed structure of iturin-A and bogorol A were elucidated by the use of nuclear magnetic resonance (NMR) (Garbay-Jaureguiberry et al., 1978; Barsby et al., 2001). Consequently, by utilizing the original primary structure as template, several analogs of iturins and bogorols were identified using mass spectrometry and reported (Rautenbach et al., 2001; Barsby et al., 2006; Zhou et al., 2020; Singh et al., 2021). Considering the role of purity of peptide for such studies, reverse-phase HPLC was used for the successful separation of unknown compounds in pure form (Figure 1B). The purified peptide showed positive reactions for ninhydrin and phosphomolybdic acid reagents in TLC analysis that established lipopeptide nature of the compound. Inhibition of growth of *S. aureus* MTCC 1430 in direct TLC bioautography confirmed the antimicrobial activity of lipopeptide. Further, destabilization of water droplets with the addition of purified lipopeptides in drop collapse assay confirmed biosurfactant nature of lipopeptide (Jain et al., 1991). MALDI analysis of the lipopeptide revealed a mass of 966.1 Da and MS/MS fragmentation pattern showed the presence of seven amino acids along with a fatty acid chain containing 15 carbon atoms. Its chemical composition is similar to iturins that typically contain heptapeptide linked to a fatty acid chain with length between C14 and C17. Likewise, amino acid composition of BLL as determined by MS/MS was partially similar to bogorol analogs (Barsby et al., 2006).

Strains of *Bacillus* and related genera are known to contain a huge repository of biosynthetic gene clusters encoding secondary metabolites with great potential for production of novel antimicrobials and detailed data are available in the antiSMASH database (Blin et al., 2017). Notably, marine isolates of *B. laterosporus* (Barsby et al., 2006) were genetically characterized to establish the biosynthesis of lipopeptides including different bogorol analogs. To confirm the production of these lipopeptides, we have analyzed the whole-genome sequences of strains GI9 and SKDU10 using the antiSMASH database. AntiSMASH analysis of strain GI9 genome sequence revealed the presence of multiple NRPS clusters encoding various lipopeptides including bogorol biosynthetic gene cluster with 100% nucleotide sequence identity. In agreement, the mass peaks observed from 1593 to 1640 Da in MALDI analysis of strain GI9 extract represented bogorol analogs (Figure 1). Additionally, two NRPS biosynthetic clusters containing all domains required for lipopeptide biosynthesis were also observed in antiSMASH analysis. One of them showed high similarity with surfactin biosynthetic cluster and the other biosynthetic cluster did not show any identity with NRPS clusters available in the antiSMASH database. Therefore, we presumed that this cluster is plausibly involved in biosynthesis of ILL.

The antiSMASH analysis of strain SKDU10 showed presence of multiple NRPS clusters including bogorol biosynthetic cluster. However, it displayed only 72% nucleotide sequence identity. Interestingly, the bogorol like NRPS cluster observed in this strain composed of standard modules involved in biosynthesis of lipopeptides comprising adenylation (A) domain responsible for activation of amino acid, and thiolation (T) domains also known as peptidyl carrier domain followed by an upstream condensation domain (C). The modules were found in arrangement to encode a 12 amino acid peptide and were terminated with a thioesterase (TE) domain, which is also known to catalyze the intramolecular cyclization (Finking and Marahiel, 2004). However, the cluster did not show the presence of all genes that are involved in bogorol production in comparison to bogorol biosynthetic cluster from *B. latersporus* strain DSM25 (Dejong et al., 2016) (Figure 2D). As a result, the lipopeptide isolated from strain SKDU10 differed in amino acid composition from that of bogorol and its analogs. Most significant difference is that it did not show characteristic Valinol residue at C-terminal, which is commonly formed in bogorols by the reduction of Val residue and the enzyme responsible for reduction was not found in bogorol biosynthetic cluster (Barsby et al., 2006). The amino acid sequence comparison of bogorols and BLL from strain SKDU10 differed in composition largely at C-terminal where Lys, Tyr, and Leu residues of bogorols were replaced with Asp, Val, and Glu signifying the genomic difference of the cluster. It is hypothesized that only 72% similarity of this cluster in strain SKDU10 with bogorol biosynthetic cluster dictated absence of genes responsible for synthesis of all amino acids in bogorols leading to differences in amino acid composition of BLL.

The MIC values observed for both the lipopeptides were high, however, BLL secreted by strain SKDU10 differed in antimicrobial activity spectrum in comparison to bogorols. BLL from strain SKDU10 inhibited the growth of fluconazole-resistant *Candida* sp. and fungal indicator strains that has not been reported previously. Similarly, ILL was found to inhibit the growth of Gram-positive and Gram-negative bacteria despite the fact that iturins have been largely reported for potent antifungal and limited antibacterial activities (Ongena and Jacques, 2008). It is pertinent to mention that the antimicrobial activity of peptides mostly depends on amino acid composition. Indeed, differences in antimicrobial activity among bogorol analogs were attributed to differences in amino acid composition (Barsby et al., 2006). Consequently, the amino acid compositional differences observed for BLL might have contribute for their biological activity against *Candida* and filamentous fungal indicator strains in comparison to bogorol that was not reported for antifungal activity. The diversity in amino acid composition is in turn directed by co-evolution of nucleotide sequence in biosynthetic gene that must have hypothetically arisen from challenges faced by producer strain in their niche/host and related antimicrobial function. As observed for bogorol analogs, BLL was also found to be thermostable in the present study. The amino acid composition of both ILL and BLL produced by strains GI9 and SKDU10, respectively, is predominated with hydrophobic amino acids. In a similar study, Zhao et al. (2012) reported an 11 amino acid antimicrobial peptide containing majority of hydrophobic amino acids indicating the significance of hydrophobic property of the peptide for its interaction with microbial cytoplasmic membrane. Accordingly, cell disruption is found to be the primary mechanism of killing by ILL and BLL as observed by EM and phase-contrast microscopy of treated cells (Figure 4).

Iturin or surfactin type of lipopeptides are well-known family of antibiotics and daptomycin is the most important lipopeptide drug with clinical applications (Chooi and Tang, 2010). The structural diversity of ILL and BLL in comparison to earlier reported lipopeptides governs their antimicrobial activity, thus allowing their usage for novel applications other than clinical therapeutics. This study documented production of iturin and bogorol like lipopeptides by strains of *Brevibacillus* that displayed antimicrobial properties. They inhibited fungal growth on tomatoes and grapes during *in vitro* experiments and did not show any phytotoxicity in seed germination assay. Lipopeptides with inhibitory activity against bacteria and fungi may be employed directly or using them as templates for improved versions. Such lipopeptides can be used to sanitize fresh produce of fruits and vegetables to eradicate deterioration and avoid wastage.

## Data Availability Statement

The datasets presented in this study can be found in online repositories. The names of the repository/repositories and accession number(s) can be found below: https://www.ncbi.nlm.nih.gov/, CAGD01000001-61; https://www.ncbi.nlm.nih.gov/, LSSO00000000.

## Author Contributions

SS, DS, SM, and SK conceived the idea. SS, SM, VG, and SK designed the experiments. SS, DS, PB, SC, Harshvardhan, and SM performed the experiments. SS, DS, PB, SC, VG, SM, and SK analyzed the data. SS, DS, SC, and SK prepared the manuscript. All authors contributed to the article and approved the submitted version.

## Conflict of Interest

The authors declare that the research was conducted in the absence of any commercial or financial relationships that could be construed as a potential conflict of interest. The reviewer DS declared a shared affiliation with the author VG, to the handling editor at the time of the review.

## Publisher’s Note

All claims expressed in this article are solely those of the authors and do not necessarily represent those of their affiliated organizations, or those of the publisher, the editors and the reviewers. Any product that may be evaluated in this article, or claim that may be made by its manufacturer, is not guaranteed or endorsed by the publisher.

## Footnotes

1
http://antismash.secondarymetabolites.org
