# Supplementary material for: Characterization and Antimicrobial Studies of Iturin-Like and Bogorol-Like Lipopeptides From Brevibacillus spp. Strains GI9 and SKDU10
Source: Front Microbiol. 2021 Oct 29;12:729026. doi: 10.3389/fmicb.2021.729026 (PMC8589628; doi:10.3389/fmicb.2021.729026)

Characterization and antimicrobial studies of iturin-like and bogorol-like lipopeptides from *Brevibacillus* spp. strains GI9 and SKDU10

Shelley Sardul Singh[†1], Deepika Sharma[†1], Piyush Baindara[1], Stanzin Choksket[1], Harshvardhan[1], Santi Mandal[2], Vishakha Grover[3], Suresh Korpole[1]*

1. CSIR-Institute of Microbial Technology, Chandigarh, India. 2. Indian Institute of Technology, Kharagpur, India. 3. Dr. Harvansh Singh Judge Institute of Dental Sciences and Hospital, Panjab University, Chandigarh, India.
[†]Both authors contributed equally; *Corresponding Author: Suresh Korpole, suresh@imtech.res.in, CSIR-Institute of Microbial Technology, Chandigarh, India

Supplementary information

Supplementary Table S1: MS/MS fragmentation pattern and error values in mass (m/z) observed for iturin like lipopeptide (ILL)

**Mass (m/z)**	**Error (ppm)**
146.7	0.53
274.2	0.74
411.1	0.19
498.9	0.2
569.1	0.34
626.3	0.46
727.6	0.09

Supplementary Table S2: MS/MS fragmentation pattern and error values in mass (m/z) observed for bogorol like lipopeptide (BLL)

**Mass (m/z)**	**Error (ppm)**
268.7	0.12
397.5	0.49
496.8	0.33
611.02	0.76
724.5	0.88
824.8	1.39
951.7	0.96
1050.7	0.44
1152.9	0.82
1263.07	0.74
1357.34	0.38

Supplementary Table S3: Effect of temperature, pH and proteases on activity of ILL and BLL

**Treatment**	**Reaction duration/condition**	**Residual activity (%)**	
		**BLL**	**ILL**
Temperature (ºC)			
37	1h (pH 7.0)	100	100
60	1h (pH 7.0)	100	100
80	1h (pH 7.0)	100	100
100	1h (pH 7.0)	100	100
121	15 min (304 kPa) (pH 7.0)	100	100
pH			
2.0	4h/ 25 °C	52	43
4.0	4h/ 25 °C	100	95.5
6.0	4h/ 25 °C	100	100
7.0	4h/ 25 °C	100	100
8.0	4h/ 25°C	100	100
10.0	4h/ 25 °C	94.3	100
12.0	4h/ 25 °C	Nil	Nil
Enzymes			
Proteinase K	6h/ 37 °C	77.7	82.3
Trypsin	6h/ 37 °C	91.6	94.1
Chymotrypsin	6h/ 37 °C	94.4	91.1
Lipase	6h/ 37 °C	100	100

Supplementary Figure S1: FTIR spectra of (A) ILL (B) BLL

A

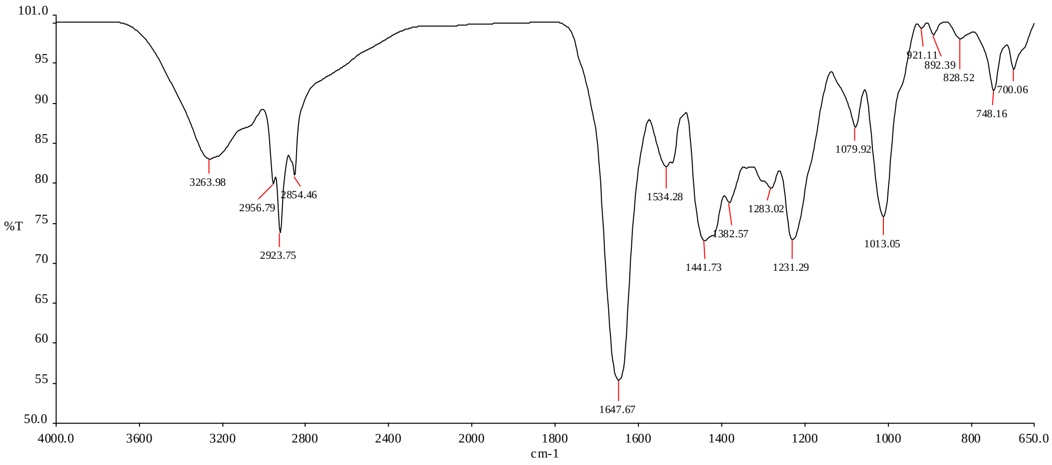

B

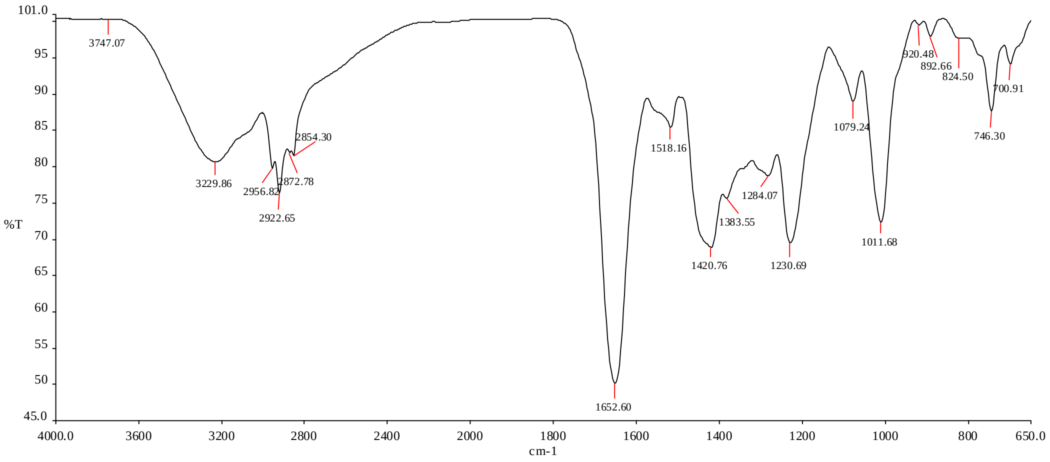

Supplement: Supplementary file 1 [file Data_Sheet_1.pdf]
